# Supplementary material for: Are carbon emissions trading and green financial instruments synergistic? -Comprehensive quantitative research based on content analysis
Source: PLoS One. 2024 Mar 7;19(3):e0298601. doi: 10.1371/journal.pone.0298601 (PMC10919601; doi:10.1371/journal.pone.0298601)
Supplement: S1 Data — (ZIP) [file pone.0298601.s001.zip › Data/地级市Z_F工作报告.zip!/║┌┴·╜¡/╞δ╞δ╣■╢√╩╨/└┤╫╘╣1⁄2╓┌║┼í╛┬φ┐╦╩2╛▌═°í┐_2014.docx]

各位代表：

　　现在，我代表市人民政府，向大会作工作报告，请予审议，并请各位政协委员提出意见。

　　一、2013年工作回顾

　　2013年，面对严峻的经济形势和严重的低温洪涝灾害，我们在市委的正确领导下，全力以赴上项目、保增长、调结构、促改革、惠民生，圆满完成了市十五届人大三次会议确定的各项任务。实现地区生产总值1230.4亿元，同比增长8.5%；财政总收入144.9亿元、地方财政收入104.6亿元、公共财政预算收入64.4亿元，分别增长18.1%、19.7%和17.1%；全社会固定资产投资894.1亿元，增长32%；社会消费品零售总额544.7亿元，增长13.4%；城镇居民人均可支配收入19064元，农村居民人均纯收入9984元，分别增长11%和13.5%。

　　——全力招商，大上项目，发展活力显著增强。重点推进固定资产投资5000万元以上产业项目237个，其中超亿元项目166个；年度完成投资315亿元，为计划的119.7%。成功引进一批战略性大项目、好项目，产业结构进一步优化。国奥生态农庄、缘子绿色食品、陈光标绿色食品产业园等项目正式签约，北京首农、佐源木糖醇、蓝天可持续建筑等项目加快建设，棒棒娃食品、百佳居陶瓷、天维大豆浓缩蛋白等项目进入设备调试阶段，北方特种合金稀土加工、鑫源特种钢锻件等项目建成投产。5个重点园区实现主营业务收入379.6亿元、利税38.1亿元，分别增长21.2%和15.1%。梅里斯绿色食品特色产业园被省批准为两大平原现代农业综合配套改革试验专业园区。依安陶瓷产业园、昂昂溪水师中小企业园、富裕农机装备制造产业园等一批特色园区发展较快。争取批次用地指标821.9公顷，项目建设用地得到保障。

　　——激活存量，做大增量，工业经济企稳趋升。规模以上工业完成增加值278.5亿元、主营业务收入949.3亿元、利税94亿元，分别增长9.5%、8%和9.4%。新增规模以上工业企业42户。27个工业项目列入省“双百工程”。北满特钢锻造改造、龙华新能源汽车及配套产业园项目部分工序建成投产。飞鹤、安泰、恒阳等食品工业企业发展迅猛，食品工业增加值占工业经济的比重达40%。县域工业增加值增长17.3%，67个项目列入全省县域“751”工程。中小企业服务中心正式运行，出资5000万元设立中小企业发展基金，重点扶持了100户小微企业。非公经济实现增加值701.3亿元，占GDP的57%。高新技术产业完成产值620亿元，增长14%。新认定国家级高新技术企业6户，齐齐哈尔重型数控机床产业集群被认定为国家创新型产业集群试点，中齐建材跻身中国中小企业创新百强，瑞盛公司成为全省首批创新型诊断服务试点企业。哈工大华工机床高新技术产业化基地开工建设。国家重载快捷铁路货车工程、省麦饭石工程等13个技术研究中心立项建设。

　　——防汛减灾，创新主体，农村经济再登台阶。粮豆薯总产270亿斤，实现“六连增”，荣获全国粮食生产先进市，五个县（市）荣获全国粮食生产先进县。新增绿色食品基地30万亩，播种面积达到1280万亩。“2513”高产创建工程核心区大垄种植面积超过200万亩，增长近1倍。奶牛存栏68.8万头，保持全省领先水平；鲜奶产量176.1万吨，增长8.7%。畜牧业实现产值191亿元，增长10%，被确定为全国农业农村信息化示范基地、全省唯一动物无害化处理长效机制建设试点城市。加大农业经营主体创新力度，农民合作社达到7622个，入社耕地2375.6万亩、农户69.6万户，分别占耕地面积的74.5%和农户总数的79.1%。流转土地1890万亩，占集体耕地的61.3%；规模经营1853.7万亩，增长37.9%。发放购机补贴9447万元，争取全省第一批现代农机合作社16个、扶持资金9700万元。田间综合机械化程度达到93.2%。兴十四现代农业园区示范带动能力进一步增强，县乡村三级农业科技园区发展到1337个。农产品加工转化率达40.5%。规划建设了18个食品产业园区，新入驻企业36户。投资7.28亿元，实施了中小河流治理、农田水利工程建设、水库除险加固等项目。新增高效节水灌溉面积23.6万亩、水田56.9万亩。投资1.9亿元，治理改良农田44.4万亩。转移农村劳动力114.8万人，实现劳务收入116亿元。

　　——繁荣市场，升级业态，第三产业蓬勃发展。万达广场、万力皮革城等新型商业业态入驻，中汇城奥特莱斯和居然之家、百花小商品商务中心、瀚沃家居建材城加快建设，铁锋现代综合物流园、中恒医药及物流园、内陆无水港顺利推进。对外贸易稳定增长，进出口总额完成8.2亿美元。成功举办绿博会、小交会等29个展会。旅游业加快发展，东北最大的动植物园开工建设，江桥抗战纪念地被授予国家4A级旅游景区，“4+1”城际旅游联盟继续深化，5家单位获省诚信旅游示范单位，旅游收入增长16.3%。与俄罗斯白山市结为友好城市。金融业健康发展，各项存款余额1293.3亿元，贷款余额908.2亿元，比年初分别增长11.7%和19.4%。哈尔滨银行、广发银行进驻营业。成功发行东北三省首单区域集优债务融资集合票据，为瑞盛、东北变压器、恒阳牛业3户企业融资1.2亿元。飞鹤原生态牧业在香港成功上市。在全省率先开展小微企业贴息贷款，发放贷款6800万元。与国家开发银行省分行签署合作协议，授信额度近60亿元。

　　——健全体系，打造精品，城乡建设快速推进。出台了3个推进“三化”建设规划框架指导意见。全市城乡建设总投资达167亿元，投入量和工程量为历年之最。新城重点建设项目31个，开工面积182万平方米，累计完成投资82亿元，5个住宅项目部分竣工并回迁，市公安指挥中心交付使用，高铁南站、第一医院南院一期、中汇城广场一期全面开工，“二纵六横”8条道路建成通车。劳动湖“通航滨水线”进出口船闸全部建成，5座跨湖桥梁全部通车。“迎宾门户线”沿街124栋楼房立面改造全面完成，整体形象明显改观。“齐扎旅游线”完成绿化11.5公顷，扎龙温泉小镇建设提速，湿地博物馆主体完工。升级改造市政道路37条、246万平方米，改造桥梁3座，改造新建污雨水管线25.4公里、供水管线18.6公里、供热管线25公里、供气管线20.7公里。齐昂公路、绥满高速卜奎互通、滨州公铁立交桥和公路客运枢纽工程进展顺利。全市开工建设棚改回迁房6078套，竣工回迁1.6万户；配建廉租房、公租房1684套；改造老旧散住宅楼518栋、405.2万平方米。改造农村泥草（危）房4.25万户。完成既有建筑节能改造242万平方米。10个省级百强试点小城镇完成投资9.8亿元，建设项目107个。城市管理“四级网络”基本实现全覆盖，拆除违规牌匾广告4393块、违章建筑2065处。物业属地化管理全面落实。入选国家智慧城市试点。

　　——注重保护，强化治理，生态建设成果丰硕。争取华电集团投资25亿元，启动3家电厂脱硫、脱硝及“上大压小”等项目建设。日处理1200吨崔门生活垃圾处理场开工建设。县（市）区污水处理厂相继建成使用，全市污水处理率达到70%。节能建筑、集中供暖能耗、“禁实”和墙改工作均处于全国先进行列。开通了首条新能源公交示范线路，淘汰黄标车2万余台，公共交通运营车辆使用清洁能源比例逐年上升。中心城区环境空气质量达标率94%，城市空气质量二级以上天数达346天。加强水源地管护，境内嫩江江段Ⅲ类水质比例达到60%。治理水土流失3万公顷，植树造林2.37万公顷；绿化升级城区38条街路、36处公园，城区绿化面积新增23.1公顷。扎龙国家级自然保护区荣获中国十大魅力湿地，9个乡镇被命名为国家级生态乡镇，拜泉县荣获国家水土保持生态文明县。14个村生态连片治理项目全面完成。万元GDP综合能耗下降5.9%。成为国家节能减排财政政策综合示范城市。

　　——统筹推进，促进公平，社会事业全面进步。新改扩建公办幼儿园29所，建设义务教育标准化学校27所。全市1180所中小学校全部实行“阳光分班”。校企合作取得新成效，职业教育工作连续8年位居全省前列。市中医院南区病房综合楼投入使用，新增国家和省级示范社区卫生中心8所，村卫生所规范化率达75%，基层医疗卫生机构全部实行基本药物制度。国家免费孕前优生健康检查项目试点实现全覆盖，当年独生子女父母奖励费全部兑现。市博物馆历史文物陈列改造工程完成，市图书馆新馆完善、马戏团大厅改造等项目进展顺利。国际鹤文化艺术节成功举办，中东铁路建筑群等4处遗址晋升国家文物保护单位，国家级历史文化名城创建工作通过专家组验收。新建全民健身工程331处，成功承办国际国内赛事8项。

　　——办好实事，增进福祉，人民生活持续改善。“一联三”民生专项行动扎实开展，民生实事得到有效落实。城镇新增就业12.32万人，其中通过机关事业单位招考、开发公益性岗位及百花集团大学生就业平台等途径，安置高校毕业生6693人。城镇登记失业率控制在3.7%。发放扶持创业小额担保贷款3.3亿元。城镇“五险”保障范围不断扩大，新农保实现全覆盖，新农合参合率达99.7%，在全省率先将失地农民纳入城镇养老保险，困难群众住院医疗救助补偿比例提高到70%，7万余名困难企业退休人员纳入医保。城乡低保对象应保尽保，保障标准分别提高到月人均376元和年人均2181元。争取财政扶贫资金1.6亿元，实施扶贫开发整村推进100个，2.65万人实现脱贫。新建100张床位以上养老机构5家，3500名残疾人得到康复救助服务。荣获全省唯一的中国爱心城市。及时下拨资金2亿元，保障了受灾群众基本生活。城区社区公益用房面积平均达750平方米，千米示范社区达到46个。农村社区建设启动率达80%，建成农村样板社区13个。富裕县塔哈镇、泰来县汤池镇划归市区管辖。实施了新一轮警务改革，天眼工程覆盖城乡重点部位，社会治安形势保持平稳。安全生产和食品药品监管成效显著，一批较难整改的安全隐患得到有效治理，被评为中国十佳食品安全城市。深入开展领导干部接访和信访积案化解年活动，解决了一批历史积案，信访形势明显好转。荣获全国法制城市创建活动先进单位。

　　——深化改革，优化服务，行政效能明显提升。行政审批“三集中、三到位”改革全面铺开，政务中心审批事项进驻率85%以上，市县乡村四级政务服务体系初步建立。市公共资源交易中心投入使用。清理规范了行政事业性收费，实行了行政执法检查审批制度，行政审批事项减少112项，精简28.6%。行政事业资产管理改革深入推进，国有资产收归政府统一管理。事业单位分类改革全面启动。食品安全监管、城市管理、司法行政体制进一步理顺。部门预算和“三公”经费公开顺利推进。自觉接受人大和政协监督，人大代表建议和政协委员提案办结率均达100%。起草审修地方性法规草案3部、政府规章草案10部。固定资产投资、领导干部离任、保障民生等重点领域审计监督不断加强。深入推进办公用房专项治理，狠刹“四风”，机关作风进一步改进。市工商局荣获第八届全国人民满意的公务员集体。

　　机关事务、外事侨务、民族宗教、新闻出版、社会科学、妇女儿童、老龄、地方志、统计、人防、民兵预备役、双拥、档案、气象、地震等工作均取得了好成绩。

　　各位代表！过去一年的成绩来之不易。这得益于省委、省政府和市委的正确领导，得益于历届政府奠定的良好基础，得益于市人大、市政协及各民主党派、社会各界的有力监督和厚爱支持，得益于驻齐部队的鼎力相助，得益于全市人民的团结奋斗。在此，我代表市政府，向全市人民，向所有为我市经济社会发展作出贡献的同志们、朋友们，向驻齐解放军、武警官兵，致以崇高的敬意和衷心的感谢！

　　在看到成绩的同时，我们也清醒地看到，全市经济社会发展和政府工作中还存在一些问题，主要矛盾仍然是发展不足。主要表现为：传统产业比重较高、新兴产业发育不足、中小企业成长缓慢、科技创新能力不强、节能减排压力巨大、县域经济实力较弱，经济发展的质量、效益、层次和可持续性亟待提高；开放程度不够，区域中心城市辐射功能不够突出，对外交流合作还需深化；城乡差距依然较大，“城市化、城镇化和城乡一体化”建设进程不均衡；各级财政惠及民生能力不强，部分城乡居民生活质量相对较低，关系群众切身利益的民生问题亟需解决；发展稳定仍面临一些体制性、机制性障碍，改革创新的任务还很艰巨；形式主义、官僚主义、享乐主义和奢靡之风现象不同程度地存在，公共资产资源利用效率不高，行政效能和公共服务能力与群众要求还有一定差距。对此，我们一定高度重视，实事求是分析，认真加以解决。

　　二、2014年工作任务

　　2014年是深入落实党的十八届三中全会精神、全面深化改革的启动之年，是推动我市经济社会加快发展、全面完成“十二五”规划的关键一年。面对新的改革创新和发展稳定任务，面对日趋激烈的区域竞争格局，只要我们认清形势，倍加珍惜十分难得的政策机遇，倍加珍惜团结向上的良好局面，倍加珍惜来之不易的发展势头，鼓足干劲，凝心聚力，创新求实，奋发有为，我市经济社会就一定能够实现快发展、大发展。

　　今年政府工作的总体要求是：深入贯彻党的十八届三中全会和中央、省委经济工作会议、城镇化工作会议精神，按照市委十二届四次全会总体部署，以“三化”为统领，以科学发展、加快发展为主题，以全面深化改革为主线，牢牢扭住经济建设不放松，突出抓好产业项目建设，把改善民生作为一切工作的出发点和落脚点，改革创新，开拓进取，促进经济持续快速发展、社会和谐稳定，为全面建成小康社会奠定坚实基础。

　　主要预期目标是：全市地区生产总值增长10%以上，公共财政预算收入增长12%以上，规模以上工业增加值增长11%以上，全社会固定资产投资增长30%以上，社会消费品零售总额增长14%以上，城镇居民人均可支配收入增长12%以上，农村居民人均纯收入增长13%以上。城镇化率提高1.5个百分点。万元GDP综合能耗下降5.9%，化学需氧量、氨氮分别削减2.7%和5.4%，二氧化硫、氮氧化物分别削减7.4%和13.2%，可吸入颗粒物(PM10)浓度降低2%，空气质量达到或好于国家二级以上（新标准）天数超过275天，耕地化肥、农药施用量均降低10%，有机、生物肥药施用量增加20%以上。

　　为实现上述目标，重点做好以下工作：

　　（一）大力推进现代农业发展，促进农业质优丰产和农民增收致富。以两大平原现代农业综合配套改革试验区建设为契机，以优化种植结构为先导，以农业产业化为龙头，以科技、农机和水利提质提效为保障，探索农业农村发展新路径。在满足全市食品加工和畜牧业发展需求的前提下，引导农民积极应对市场变化，压缩玉米、大豆种植面积，全面扩大水稻、杂粮、马铃薯种植面积。其中，在完善水田发展规划基础上，今年新增水田面积60万亩以上。新建国家级绿色食品原料基地120万亩，新增省级龙头企业3户、市级龙头企业10户，产业化龙头企业主营业务收入增长10%以上。积极引进优良品种，发挥农业科技园区示范带动作用，推广高产栽培技术模式。深入实施“2513”高产创建工程，核心区大垄高产栽培达到340万亩。以农机合作社建设为切入点，创新农业生产经营主体，发展多种形式、多种类型的农民合作社，加快土地规范有序流转，促进土地规模经营，提高种植水平和效益，实现粮食增产提质，并探索创建高级合作社的新模式，向产业化方向发展。加强农田水利基础设施建设，改善除涝面积114万亩。实施中小河流治理项目12项，消险加固小型水库3座。抓好音河、卫星灌区等项目建设。扶持棚室蔬菜生产，建设区域中心蔬菜基地，新建标准化设施蔬菜小区3个，蔬菜种植面积达到71万亩。大力发展畜牧业，突出抓好规模标准养殖、增量提质，新建存栏2000头现代示范奶牛场16个、市级奶牛和肉牛产业试验示范园各1个、县级畜牧养殖园区10个以上，促进飞鹤、光明等现代示范奶牛场如期达产达效。奶牛存栏70万头，鲜奶产量达到185万吨。实施农村土地综合治理16万亩，扶贫开发整村推进99个。转移农村劳动力117万人，实现劳务收入120亿元。

　　（二）打好产业项目攻坚战，增强区域经济可持续发展能力。把产业项目建设作为加快发展的重要引擎，发挥资源和区位两大优势，依托资源和市场上项目，使项目建设在数量、质量、规模和层次上实现新突破。对已签约项目抓开工、已开工项目抓完工、已完工项目抓运行、已运行项目抓达效，确保新开工、竣工及达产项目数量均超过去年。全力推进260个以上固定资产投资超5000万元的产业项目。加快朗盛陶瓷、海亮苏打水等34个在建项目建设，促进北京首农、棒棒娃食品、百佳居陶瓷等58个项目达产达效。坚持走出去、请进来，加大内引外联力度，依托存量引进增量。通过存量裂变生成项目、服务好项目以商招商、谋划好项目主动招商、围绕产业链条延伸招商、围绕主导产业及龙头企业配套集聚招商等措施，实现招商引资新突破。在坚决守住不破坏生态环境、不上产能过剩项目、不突破政策法律三条底线的基础上，注重项目纳税和吸纳就业的可持续性，实现政府与企业互惠、经济效益和社会效益双赢。加快产业园区建设，以高新区为牵动，积极发展一批特色鲜明的专业化园区。创新园区建设模式，适度超前搞好基础设施建设，不断提升承载能力，吸引优质项目快速向园区集中。争取富拉尔基经济开发区晋升为国家级经济开发区，讷河、富裕、依安、龙江等园区晋升为省级园区，梅里斯绿色食品特色产业园成为省级工业示范园区。严格管控土地利用，做好土地收储及利用的各项衔接，确保土地利用实现集约化、高效益、可持续。

　　（三）以优化结构为主线，促进工业经济提速增效。紧紧抓住国家实施新一轮老工业基地调整改造规划的契机，加快工业经济转型升级。对“老字号”企业加大扶持力度。巩固传统产业基础地位，为企业创造优良的服务环境、生产环境和宜居宜业的人文环境。支持一重、北钢、齐轨交通、齐重数控、齐二机床、昊华等重点企业技术改造、新产品研发和市场应对，促进装备制造、冶金、化工等传统产业技术和产品升级。装备制造业主营业务收入增长10%以上。对“原字号”资源进行深度开发。大力发展农副产品精深加工业，将我市的资源优势转化为产品优势和经济优势。加快发展水、乳、豆、薯、酒等九大产业，特别是充分利用我市酒产业资源，积极引进市场龙头企业和战略投资者，迅速壮大酒产业。加大国奥生态农庄、缘子绿色食品和陈光标绿色食品产业园推进力度，支持北大仓、富裕老窖、飞鹤、安泰、元盛等食品企业做强做大。农产品加工转化率达到42%，精深加工比重达到29%。食品加工业主营业务收入增长20%以上。对“新字号”产业进行培育壮大。强化科技支撑和金融服务，推动产、学、政、研协同创新，促进企业快速成长。重点推进新能源汽车、金属基复合材料、高新技术产业化基地、中恒医药及物流园、清华同方信息港、稀土合金研发中心等项目建设。深入研究制定生物产业发展规划，为项目生成落地做好基础性工作。高新技术产业实现产值680亿元以上，增加值占GDP的10%以上。全力推进县域经济走特色发展之路，坚持“一县（区）一品、一品多县（区）、一县（区）多品”，对“一品多县（区）”的，全市搭建平台，统一整合，共同打造，实现优势产业竞相发展、集群发展、协调发展。

　　（四）强化引导服务，推动民营经济全面快速发展。本着“非禁即入”的原则，毫不动摇地鼓励、支持和引导非公有制经济快速发展，民营经济增加值增长20%以上。鼓励全民创业，着力解决创业环境不优、创业信息不畅、创业金融支持不强等问题，营造尊重创业、尊重创造、尊重创新的良好氛围。充分发挥行业协会和中小企业服务中心作用，积极引导中小企业适时进行股份制改造，充分发挥齐大、哈工大等市内外高校及科研院所的优势，建立生产经营联盟和科技创新联盟，解决好中小企业在成长中遇到的技术和管理难题，尽快把中小企业做实、做精、做强。认真落实国家和省市的相关政策，对成长性好的中小企业，在产品升级和技术改造方面给予土地、税收、金融等政策支持，年内争取40户以上中小企业进入规模以上工业企业行列。积极引导民营企业建立科学的管理和运营机制，提高民营经济发展水平。加强对民营企业创办者、经营者的培训，提升民营企业家队伍整体素质。拓宽民营企业融资渠道，大力发展直接融资，争取第二期区域集优债务融资、首批中期票据和第三期企业债券发行。积极推动民营企业上市，使其在资本市场及金融环境中获得更大的支持。

　　（五）完善区域中心城市辐射带动功能，加快发展现代服务业。积极发展中汇城、万达广场、万力皮革城等新型商业业态，力促百货大楼、百花小商品商务中心等传统商业转型升级，大力发展电子商务、连锁经营、物流配送等现代流通方式，引导和规范分散经营的物流企业尽快入驻规模化的综合物流园区，加快构建辐射周边2000万人口的新型“商圈”及物流基地。加快航空口岸建设，加大通往珠三角、长三角中心城市航线密度。借助我省和内蒙古东北部地区沿边开发开放规划的实施，发挥铁路、公路交通优势，依托满洲里口岸，发展对俄、对欧贸易。巩固扩大与澳大利亚、新西兰、日本、韩国友好城市的交流合作，重点引进项目和资金。争取在美国、德国、法国等国家和俄罗斯腹地缔结新的友好城市，加快产品对外输出。办好绿博会和小商品、种薯、杂粮、肉牛等交易会，创办家具、工业配件等专业展会及苏打水健康论坛，推动本地产品走出去，创出品牌。整合区域旅游资源，优化旅游市场功能定位和产品组合。提升扎龙湿地、温泉小镇、劳动湖、动植物园、江桥抗战纪念地等景区景点品位，精心打造生态农庄假日游、湿地观鹤游、冰雪游等特色旅游文化品牌，进一步丰富“4+1”城际旅游联盟精品路线，旅游收入增长15%以上。在注重我市历史文化积淀与俄罗斯建筑文化元素相结合，注重文化与旅游、时尚、娱乐产业相结合的基础上，探索我市历史文化的保护开发利用之路。大力发展文化创意、咨询及设计产业，为企业提供配套服务，打造新的经济增长点。继续引进外埠金融机构，规范整合现有小额贷款公司，逐步构建开放、有序的大金融格局。加强对各类融资担保公司、小额贷款公司监管，防范金融风险。鼓励金融机构优化信贷结构，扩张信贷总量，提高存贷比，加大支持实体经济力度。

　　（六）以“三化”建设为统领，加快城乡建设提档升级。坚持生态优先、交通优先、就业优先，加快编制“三化”建设规划。做好劳动湖与嫩江互通水系全线规划及景观设计，搞好联通大道、卜奎北大街两侧重点区域规划设计。以突出鹤文化特征为重点，做精城市景观规划设计，增强城市的美感和文化内涵。推进城乡“三供两治”和市政道路等基础设施建设。市区重点改造5座污雨水泵站，新建污雨水管线8.9公里，完成北大桥、滨湖桥供热燃气管线迁移，实施二次供水加压泵站应急抢修，加快区域集中供热中心建设，推进崔门生活垃圾处理场、医疗废物处理、污泥无害化处置和城镇污水厂等项目建设和完善。中心城区新建、延伸主次干道15条。齐昂公路、绥满高速卜奎互通、滨州公铁立交桥交工通车。全面完成哈齐高铁运行各项前期工程，公路客运枢纽主体完工并与高铁南站同步交付使用。优化住房结构，建立保障房、商品房互为补充的多层次住房供应体系，满足不同群体的住房需求。以创建国家级园林城市为目标，完成中心城区绿地修建性规划，采取裸地覆绿、拆墙透绿、见缝插绿、大树进城和公园建设等方式，新植树木117万株，新增绿地18公顷，凸显“公园城市、城市公园”特色。全市植树造林1.67万公顷。加大湿地保护力度，扎实做好扎龙湿地核心区移民工作。以推进国家节能减排财政政策综合示范城市建设为契机，突出生态建设，严格控制PM10和PM2.5等主要大气污染物排放，加强重点工业企业、城区原煤烟囱、机动车尾气等污染源治理，实施“公交都市”战略，推广节能和新能源汽车。实施主道、巷道、小区和楼道全面亮化，依法依纪拆除违章建筑，规范建筑工地，实施机械化清扫、清运、清雪，努力让城市更加明亮、更加整洁，实现人民城市人民管、人民城市人民爱。加强城乡环境综合治理，重点改善乡容村貌，确保乡风文明、村容整洁、道路通畅、出行方便。

　　（七）切实办好民生实事，努力增进群众福祉。坚持民生为基、民生优先，守住底线、突出重点，从群众最不满意的地方改起，从群众最需要的地方做起，突出抓好33件民生实事。（1）加快保障性住房建设，市本级改造棚户区3030户，竣工回迁房8000套；建设公共租赁住房400套、2.2万平方米。（2）完成卜奎南大街、301国道及中心城区57条无灯巷道亮化工程，并改善提高中心城区主次干道亮化程度。（3）推进供热设施升级改造，启动3座区域热源建设，撤并整合市区5吨以下小锅炉39台，改造中心城区老旧供热管网77公里。（4）完成劳动湖水系12座桥梁建设并实现全部通车。（5）改善中心城区居民通行环境，改造巷道35条,占需改造的50%；硬化或绿化裸土地面10万平方米；修复破损路面10万平方米，更换破损步道砖2.8万平方米。（6）市本级完成既有建筑节能改造100万平方米。（7）启动嫩江中心城区段规划设计和沿江休闲及景观带建设。（8）推进新城重点工程建设，第一医院南院一期工程、市中级法院审判综合楼主体完工，居然之家、五金建材城实现商家入驻，启动新城体育场规划建设。（9）综合整治中心城区204个住宅小区的违章建筑，同步规范小区秩序。（10）提高环卫机械化作业率，中心城区机械化清扫率和洒水率均达到60%以上，生活垃圾全部实现袋装化和机械化运输。（11）中心城区14个公园全部实现亮化和拆墙透绿，并完善园中绿化，配齐健身设施。（12）在中心城区主干道两侧新建水洗或环卫公厕40座；对所有街路绿地超高土全部进行清理。（13）解决中心城区312栋弃管楼的楼道亮化、门窗修缮和卫生管理问题。（14）搞好城市公交发展规划及网络布局，整合公交资产资源，新增新能源公交汽车70台，建设标准化公交站亭370处。（15）实施就业再就业工程，新增就业10万人，城镇登记失业率控制在4.5%以内；发放小额担保贷款4亿元；完成再就业职业技能培训4万人、创业培训1万人、劳务输出前培训3万人。（16）推进社区标准化建设，新增5个1000平方米以上城市示范社区公益用房，完成10个500平方米以上农村样板社区公益用房建设。（17）新建5家100张床位以上的民营养老机构。（18）提高城乡低保标准，确保资金足额发放到位。（19）将低保和低收入失能、半失能老人护理补助标准提高一倍。（20）规范理顺社区工作人员管理体制，提高社区工作人员工资待遇，两年达到省要求标准；提高中心城区环卫工人工资标准。（21）实施中小学生及幼儿眼睛关爱工程，为全市中小学和幼儿园教室安装更换护眼灯。（22）完成181所农村学校取暖设施改造，全部取消小火炉。（23）健全农村医疗保障体系，农民参合率保持在99%以上，统筹区域政策补偿比例达到75%以上，全部实行先住院后结账。（24）健全四级卫生服务网络，实现乡村、社区全覆盖；今年将全市50%的乡镇卫生院纳入县乡一体化管理，乡村医疗机构一体化管理率提高到85%；9家县医院全部与三级医院建立医疗协作关系，在心脑血管疾病、恶性肿瘤等方面开展深度合作，降低群众转诊、转院率，减轻群众就医负担。（25）设立500万元文化发展基金，支持文艺创作和文化产业发展。（26）整合市科技企业孵化器及创业基地，在高新区建立市级统一的科技研发中心科技企业孵化器，并争取晋升为国家级孵化器。（27）建设100套中小企业科技人员安家公寓。（28）开展全民健身运动，创编并推广鹤城健身操，新建全民健身场所120处，安装各类健身器械720件。（29）实施残疾人关爱工程，为升入高等院校的特困残疾人考生及贫困残疾人家庭考生给予一次性助学补贴，为630名以上贫困精神病患者提供免费住院与药物救助服务，为180名以上0-6岁各类贫困残疾儿童提供康复救助服务。（30）加强食品安全监管，建立市、县（区）、街道、社区和食品安全网格的“五级网络、四级平台”食品安全监管体系，实现食品安全监管无盲区、全覆盖。（31）整合涉及公共卫生、自然灾害、事故灾难、城市建设与管理、生产和社会安全等专业平台，建立全市统一的应急管理平台，实现市区监控管理全覆盖。（32）增加260万元见义勇为基金，使基金总额达到500万元，重奖见义勇为人员。（33）启动工人文化宫翻建工程，完成规划设计并开工建设。

　　（八）加强政府自身建设，打造人民满意政府。推进全市经济社会快速发展，必须着力建设为民、务实、清廉政府。我们政府全体公务人员务必牢记党的根本宗旨，把对党的事业和全市人民的忠诚转化为强烈的进取心、责任感和奋发有为的工作状态。坚持把改革创新、转变职能贯穿于政府工作始终。深入推进行政审批“三集中、三到位”改革，完善公共资源交易中心、政务服务中心、便民服务体系功能，建立中介服务超市，简化审批程序，优化审批流程，强化绩效问责，提高工作效率。继续清理、取消、下放行政审批事项，最大限度向市场、社会、基层放权，减少政府对资源配置的直接干预。抓好行政执法体制改革，促进高效执法、阳光执法。加大政府购买公共服务力度，让事业单位充满活力、社会事业蓬勃发展。整合职业教育资源，将职业教育办出特色，为各项事业发展提供技能人才支撑。完善国有资产及国有资本经营预算制度，提高国有资本收益上缴公共财政比例。统筹推进集体土地、农村金融、国有粮库、社会治理、医疗卫生、文化体制、就业体制等各方面改革。建立政府重大问题和事项集体决策、专家咨询、社会公示听证、决策评估等制度体系，实现科学民主决策。加大政务公开力度，推进部门预算、行政收费和执法检查网上公开，增强政府工作透明度。切实加强诚信政府、法治政府建设，做到依法行政、重信守诺，不断提高政府公信力。自觉接受人大依法监督、政协民主监督、媒体监督、公众监督，让各界对政府工作有充分的知情权、参与权和监督权。认真开展党的群众路线教育实践活动，落实中央“八项规定”、省委“九项规定”和市委实施办法，坚持开源节流，坚决反对“四风”，严控“三公”经费，降低行政成本，把有限的财力更多地投向民生。严格执行党风廉政建设责任制，认真落实“一岗双责”，强化对重点行业、重点项目的监察和审计，严防腐败现象发生，切实做到干部清正、政府清廉、政治清明。

　　各位代表！齐齐哈尔正处在经济社会转型升级和加快发展的关键时期。面对新的使命，我们深感责任重大。让我们在市委的正确领导下，紧紧依靠全市人民，鼓足干劲，改革创新，凝心聚力，奋勇拼搏，确保圆满完成全年各项目标任务，为加快推进“三化”建设、全面建成小康社会、创造齐齐哈尔更加美好的明天而努力奋斗！
